# Research protocol for a mixed-methods study to characterise and address the socioeconomic impact of accessing TB diagnosis and care in Nepal

**DOI:** 10.12688/wellcomeopenres.15677.2

**Published:** 2020-06-16

**Authors:** Kritika Dixit, Bhola Rai, Tara Prasad Aryal, Gokul Mishra, Noemia Teixeira de Siqueira-Filha, Puskar Raj Paudel, Jens W. Levy, Job van Rest, Suman Chandra Gurung, Raghu Dhital, Olivia Biermann, Kerri Viney, Knut Lonnroth, S Bertel Squire, Maxine Caws, Tom Wingfield

**Affiliations:** 1 Birat Nepal Medical Trust, Lazimpat Road, Lazimpat, Ward No 2, Box 20564, Kathmandu, Nepal; 2 Social medicine, Infectious diseases, and Migration (SIM) Group, Department of Public Health Sciences, Karolinska Institute, Solnavägen 1, 171 77 Solna, Stockholm, Sweden; 3 Departments of International Public Health and Clinical Sciences, Liverpool School of Tropical Medicine, Pembroke Place, Liverpool, L3 5QA, UK; 4 KNCV Tuberculosis Foundation, Postbus 146, 2501 CC Den Haag, The Netherlands; 5 Tropical and Infectious Disease Unit, Liverpool University Hospitals NHS Foundation Trust, Prescot Street, Liverpool, L7 8XP, UK

**Keywords:** Tuberculosis, poverty, catastrophic costs, socioeconomic support, social protection, healthcare access, Nepal

## Abstract

**Background: **WHO’s 2015 End TB Strategy advocates social and economic (socioeconomic) support for TB-affected households to improve TB control. However, evidence concerning socioeconomic support for TB-affected households remains limited, especially in low-income countries.

**Protocol: **This mixed-methods study in Nepal will: evaluate the socioeconomic impact of accessing TB diagnosis and care (Project 1); and create a shortlist of feasible, locally-appropriate interventions to mitigate this impact (Project 2). The study will be conducted in the Chitwan, Mahottari, Makawanpur, and Dhanusha districts of Nepal, which have frequent TB and poverty.

The study population will include: approximately 200 people with TB (Cases) starting TB treatment with Nepal’s National TB Program and 100 randomly-selected people without TB (Controls) in the same sites (Project 1); and approximately 40 key in-country stakeholders from Nepal including people with TB, community leaders, and TB healthcare professionals (Project 2).

During Project 1, visits will be made to people with TB’s households during months 3 and 6 of TB treatment, and a single visit made to Control households. During visits, participants will be asked about: TB-related costs (if receiving treatment), food insecurity, stigma; TB-related knowledge; household poverty level; social capital; and quality of life.

During Project 2, stakeholders will be invited to participate in: a survey and focus group discussion (FGD) to characterise socioeconomic impact, barriers and facilitators to accessing and engaging with TB care in Nepal; and a one-day workshop to review FGD findings and suggest interventions to mitigate the barriers identified.

**Ethics and dissemination: **The study has received ethical approval. Results will be disseminated through scientific meetings, open access publications, and a national workshop in Nepal.

**Conclusions: **This research will strengthen understanding of the socioeconomic impact of TB in Nepal and generate a shortlist of feasible and locally-appropriate socioeconomic interventions for TB-affected households for trial evaluation.

## Introduction

Tuberculosis (TB) disease, which kills 1.5 million people annually, is driven by poverty ^1^. Having TB disease can also worsen impoverishment through loss of income and costs of accessing care ^2– 5^. Such costs can become “catastrophic”, leading patients to abandon treatment, develop drug-resistance, and die ^6^. WHO’s 2015 End TB Strategy advocates elimination of catastrophic costs and provision of socioeconomic support for TB-affected households ^5^. Nevertheless, there is minimal evidence concerning the ideal interventions to realise this policy change ^6– 10^. This research will generate preliminary evidence to fill this knowledge gap in a low-income country: Nepal. The findings will inform a randomised controlled trial of socioeconomic support for TB-affected households in Nepal.

**Figure 1.**
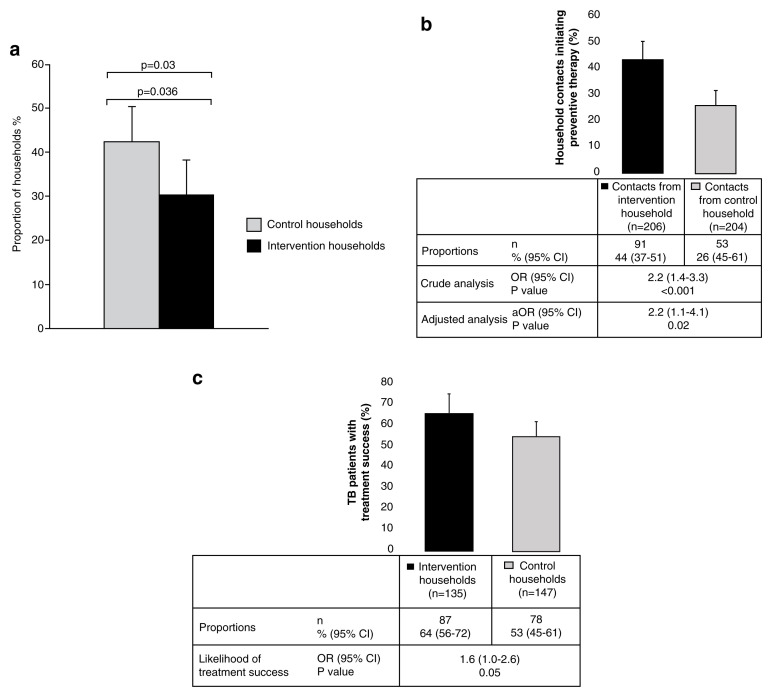
**a**) Catastrophic costs incurred by intervention (n=135) and control (n=147) TB-affected households of Callao, Peru. **b**) Preventive therapy initiation in household contacts of intervention (n=206) and control (n=204) TB-affected households of Callao, Peru. **c**) TB treatment success in patients from intervention (n=135) and control (n=147) TB-affected households of Callao, Peru. Part **a**) has been reproduced with permission from Wingfield *et al.* ^13^ Parts **b**) and **c**) have been reproduced with permission from Wingfield *et al.* ^15^.

Research from Peru has demonstrated that the severe socioeconomic impact of TB can lead to catastrophic costs (defined by WHO as >20% of a household’s annual income) ^6^. The same team also demonstrated that socioeconomic interventions for TB-affected households can mitigate catastrophic costs ( Figure 1a), improve TB preventive therapy uptake ( Figure 1b), and increase TB treatment success ( Figure 1c) ^11– 15^.

However, despite these encouraging findings, Peru is a middle-income country with a strong TB program and existing national cash-transfer schemes. To make this research replicable and applicable in diverse settings, interventions similar to those in Peru need to be adapted to other country contexts, especially low-income countries (LICs) with less developed social protection schemes and high TB burden. One such country is Nepal.

The estimated incidence of TB in Nepal is 154/100,000 people with 45,000 cases of TB being notified. Amongst these cases, there were 6,800 TB-related deaths. Multi-drug resistance (MDR) rates were 2.2% in new cases and 15% among retreatment cases. Importantly, despite good treatment success rates overall of more than 90% reported in Nepal, there remain shortcomings in TB care: treatment success rates were 70% for patients with MDR-TB and only 9% for those with HIV-TB co-infection; and accessibility of TB care remains low with treatment coverage of 70% ^16^.

The poor treatment coverage in Nepal may, in part, be due to the financial impact on TB-affected households, which is estimated to be high ^17^. Responding to this estimated burden, the Nepal National TB Programme (NTP) national strategic plan for 2016–2021 identifies provision of a support package to TB-affected households (specifically targeting MDR-TB-affected households) as a priority aim for the country with the goal of reducing catastrophic costs. There is currently limited evidence concerning the potential acceptability, impact, or cost-effectiveness of such a package, with which to inform and guide this policy decision. A cohort study has suggested that, during their illness, TB patients in Nepal experience decreased income and the total costs of accessing free TB treatment equate to nearly one quarter of annual household income ^17^. Further studies in Nepal have shown that clinic fees make up the largest proportion of direct costs and that TB patients who are poorer, are migrants, or are from rural areas, experience a disproportionate burden of total costs ^18– 20^. It is vital that vulnerable TB patient households most at risk of incurring TB-related costs are identified in order to prevent and cure TB and mitigate further impoverishment and its consequences. In non-randomized studies, incurring higher TB-related costs and not receiving education about TB have both been found to be associated with worse TB treatment adherence and adverse TB treatment outcomes ^18, 21^. Therefore, the financial impact of having TB disease in Nepal constitutes a challenge to achieving TB control and elimination.

To date, no study of TB-affected households’ costs in Nepal has been performed using the standardised global methodology for measuring costs related to accessing and engaging with TB care: the WHO TB Patient Costs Survey ^22, 23^. The studies above collected data at only one time point rather than repeated time points (e.g. cross-sectional rather than longitudinal) and did not robustly analyse the socioeconomic position, nutritional status, coping strategies, or linkage to social protection of TB-affected households. There have been no trials of socioeconomic support for TB-affected households in Nepal but formative qualitative analysis and a non-randomized pilot interventional study (offering education and financial support to patients with MDR-TB) suggested improved treatment outcomes ^24^.

However, studies reporting quantitative data alone will be insufficient to influence and change policy. Interaction with and among stakeholders has been described as the key facilitator for knowledge translation and evidence-informed health policymaking ^25^. These interactions can include policy dialogues between stakeholders to deliberate on a priority topic. Therefore, complementary to quantitative data, the focus group discussions (FGDs) and workshop detailed within this protocol will aim to expand a policy dialogue on socioeconomic support for TB-affected households among key stakeholders in Nepal, from people with TB to community leaders to TB healthcare professionals. Policy dialogues represent knowledge translation to support the integration of research evidence with tacit knowledge of local health policy-makers to: inform future policy decisions in often complex and dynamic contexts; and foster proactive collaboration ^26, 27^. These elements will be critical for successful implementation of any future interventions ^28^.

In summary, this mixed-methods research will complement and extend the existing knowledge base on social determinants and consequences of TB. More specifically, the culmination of the research will be the creation of a shortlist of feasible and locally-appropriate socioeconomic interventions for TB-affected households for future randomized controlled trial evaluation in Nepal.

## Protocol

### Ethical statement

Ethical approval was granted from the University of Liverpool, UK, research ethics committee in April 2018 (approval number 2436) and then the National Health Research Council of Nepal (NHRC) research ethics committee in May 2018 (approval number 320/2018).

Participant information leaflets will be provided and written informed consents will be obtained from all study participants for Project 1 Interviews (separate consent forms and information leaflets for patients and healthy controls), Project 2 Surveys, FGDs, and the workshop. These documents are available in the Extended Data section attachments ^29^.

All medical records obtained from the Nepal NTP will be kept confidential. Practically, through liaison with NTP Project Staff (as already organised for IMPACT-TB), the PM, PI, and RA will photocopy patient records from the Nepal NTP TB register obscuring the patient’s identifiable details. Photocopies will be marked with that patient’s unique study number identifier. No individual patients will be identifiable from publications resulting from this study.

### Study design

This mixed-methods study was funded by a Wellcome Trust Seed Award in Science (awards provided to early-career researchers to develop a novel idea that will go on to form part of a larger grant application) and will be divided into two complementary projects. Project 1 consists of a cohort study characterising the socioeconomic impact of TB on TB-affected households and a nested case-control study examining the social determinants of TB. Project 2 consists of a mixed quantitative-qualitative cross-sectional study using surveys, FGDs and a workshop to identify the barriers and facilitators to accessing and engaging with TB diagnosis and care in Nepal and suggesting potential interventions to mitigate the socioeconomic impact and improve access and engagement. The study will take place within the infrastructure of the larger EU-Horizon 2020 funded “IMPACT-TB” project, which is a study evaluating proven TB active case-finding (ACF) interventions in Nepal and Vietnam (grant 733174, http://www.impacttbproject.org/).

### Primary aims

The primary aim of Project 1 is to evaluate the socioeconomic impact on TB-affected households of accessing and engaging with TB diagnosis and care in Nepal and compare that impact in people with TB identified through standard passive case finding (PCF) versus ACF. The primary aim of Project 2 is to collaborate with key stakeholders in Nepal to create a shortlist of potentially feasible and locally-appropriate socioeconomic interventions to mitigate this impact.

### Secondary aims

The secondary aim of Project 1 is to compare the social determinants of TB (including socioeconomic position, housing situation, knowledge about TB, comorbidities, quality of life, food security, and social capital) in people with TB versus people without TB from the same districts. The secondary aim of Project 2 is to collate the opinions of key stakeholders from diverse sectors about barriers and facilitators to accessing and engaging with TB care in Nepal.

An additional aim across the study is to generate a policy dialogue and form a collaborative research network to support development and implementation of a future randomised control trial of socioeconomic support for TB-affected households in Nepal.

### Study setting

**Figure 2.**
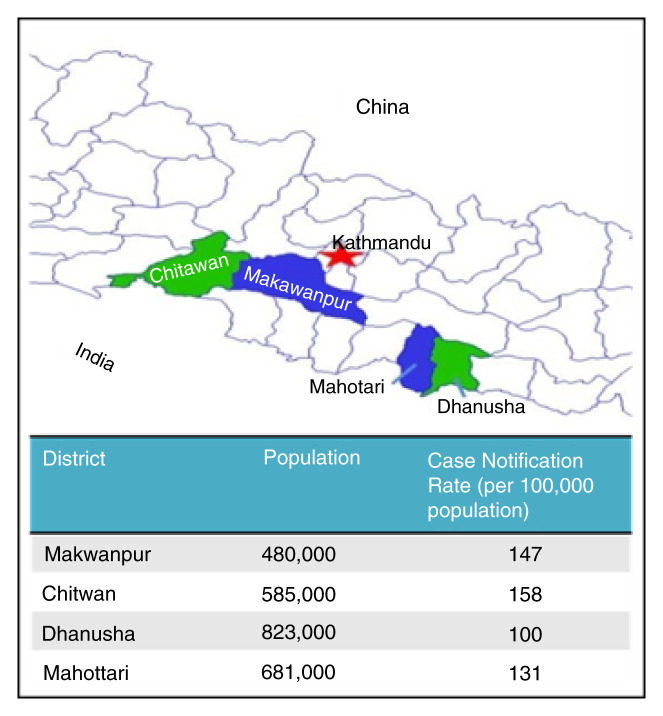
Map, population, and TB case notification rate of the four study site districts. Data for population and TB cases notification rate shown is taken from the Nepal TB Centre 2018 report. The four study site districts are labelled and highlighted in blue and green.

The study will take place within the infrastructure of the larger IMPACT-TB study, which works with a well-established international non-governmental organisation, Birat Nepal Medical Trust (BNMT), to implement ACF activities including sputum-microscopy camps and roll-out of GeneXpert OMNI in four intervention and two control districts (with PCF only). The four districts are located in the central development region of Nepal and were selected for the IMPACT-TB project based on comparable populations and TB case detection rates ( Figure 2).

The primary outcome of IMPACT-TB is the effect of ACF on early TB case detection and the study does not involve support packages for TB-affected households during treatment. This presents a unique opportunity for the complementary research described in this protocol to add value to IMPACT-TB, explore the socioeconomic impact of accessing TB care, and shortlist locally-driven strategies to reduce this impact.

### Study population

***Participant identification, recruitment and follow-up***. During Project 1, approximately 200 people with TB (cases) consecutively recruited to Work Package 3 (WP3) of IMPACT-TB (Health Economic Analysis) in the four study sites will be further recruited to this study. 100 of these cases will have been diagnosed through ACF and 100 diagnosed through PCF. Whilst attending the NTP TB clinic, these people with TB will be invited to participate in this mixed-methods research with a separate written, informed consent (see Project 1 Interview Patient Consent Form, *Extended data*) ^29^. During months three and six of their TB treatment, household visits will be conducted by BNMT project staff including community mobilisers.

Concurrently, 100 people without TB from the study sites (controls) will be invited to participate with written, informed consent (see Project 1 Interview Healthy Control Consent Form, *Extended data*) ^29^. Due to constraints in study budget, duration, and field logistics, it will not be possible to age and sex match cases and controls nor to randomly select controls using geospatial or other household randomisation techniques. Therefore, a convenience sampling strategy was used. In order to be as widely representative of and generalisable to the background population as possible, following project team and community mobiliser discussions in the study sites, we opted to recruit control participants from diverse locations within the districts, which were attended by a broad demographic cross-section of the local population in terms of age, gender, and socioeconomic position. These diverse study site locations include: tea houses, primary healthcare centres, antenatal and immunization clinics, door-to-door visits following sputum camps (e.g. people who tested negative for TB), and public gathering places. Interviewers will visit the control recruitment locations at similar times of the morning and aim to consecutively recruit all individuals in attendance at that location. Similar numbers of participants from each recruitment location will be recruited until the sample size of 100 people without TB is reached. We acknowledge that convenience sampling may be associated with a higher likelihood of a non-generalisable control cohort than other techniques. To try to address this, the survey used to collect data from the controls has incorporated multiple questions concerning sociodemographic variables, including education level, occupation, amenities, and assets, from the most recent version of the Nepal Household Survey, which is publicly available. Descriptive analysis will evaluate whether the controls recruited are representative of the wider population in those districts through comparison of their sociodemographic data with respondents to the national survey in the same districts. Any differences between the control population and background population will then be highlighted transparently in corresponding research outputs and publications.

Project 1 inclusion criteria for Cases included: being a person with TB notified to the NTP and recruited to WP3 of the IMPACT-TB study; being aged 18 years or above; and giving verbal and written informed consent to participate. Project 1 exclusion criteria for Cases included: being under 18 years of age; being a person with TB not notified to the NTP and/or not recruited to WP3 of the IMPACT-TB study; being a person with TB notified to the NTP but with a recorded domiciliary address outside of the study site districts; and being unable or unwilling to give written and/or verbal informed consent to participate.

Project 1 inclusion criteria for Controls included: being 18 years or above with primary residence in the study site communities; not currently known to be a person with TB or have a member of the household currently known to be a person with TB (e.g. not diagnosed or notified or receiving TB treatment); and giving verbal and written informed consent to participate. Project 1 exclusion criteria for Controls included: being under 18 years of age; not having primary residence in the study site communities; known to be a person with TB or have a household member with TB currently (e.g. diagnosed and/or notified and/or receiving TB treatment); and being unable or unwilling to give verbal and written informed consent to participate. 

To generate the population for Project 2, a literature review and desk-based scoping review will identify a list of key in-country stakeholders from Nepal from diverse groups including: civil-society representatives; community leaders; and TB healthcare professionals including NTP managers and multi-disciplinary staff. Approximately 50 stakeholders will be selected through purposive sampling and invited to participate in: a pre-FGD survey, FGD, and a one-day workshop. A subset of purposively sampled TB patients recruited to Project 1 (including those with multi-drug resistant TB) will be among the stakeholders invited to participate.

Project 2 inclusion criteria include: being aged 18 years or above; belonging to a stakeholder group as defined above and/or identified during scoping exercise; and being able and willing to provide verbal and written, informed consent. Exclusion criteria for Project 2 are not meeting the inclusion criteria and/or being a person with TB who has not yet taken two weeks of TB treatment or is otherwise considered to still be infectious (e.g. MDR-TB with positive sputum smear or culture).

***Sample size and statistical power***. The sample size for Project 1 is approximately 200 Cases recruited to WP3 of the IMPACT-TB project and 100 Controls from the four study site districts. This sample size is opportunistic and pragmatic: related research suggests that data from 100 people with TB gives a representative spread of costs for a given context ^30, 31^.

For Project 2, each FGD will consist of approximately eight stakeholders. The estimated number of FGDs at which information power (or saturation level) will be reached is six ^12^. Thus, the sample size for Project 2 is approximately 40 stakeholder participants. We will invite 55 stakeholders as a contingency because we anticipate an attrition rate of 20–30% during the course of Project 2.

Given that this Seed Award research will be exploratory, preparatory (e.g. for the future trial), and does not include an intervention, no calculations of statistical power are required.

### Study interventions

The study will not include any interventions or require any patient samples (e.g. blood / sputum / tissue).

### Study activities

Broadly, the activities involved in the research will include interviews with Cases and Controls during household visits, and a pre-FGD survey, FGD, and workshop with key stakeholders.

During Project 1, the project team will support BNMT district coordinators, community mobilisers, and community volunteers to do household visits to approximately 200 Cases with TB (100 diagnosed through ACF and 100 through PCF) recruited to WP3 of the IMPACT-TB project. People with TB will receive two household visits, the first during month three of TB treatment and the second visit during month six of TB treatment (to correspond with treatment completion). For controls, a single household visit and interview will be done. The interviews will be structured (see Project 1 Interview, *Extended data*) ^29^ and gather data on: i) socioeconomic position, evaluated by a multi-dimensional poverty score ^6, 9, 13, 15^ assessing dwelling characteristics, assets, and access to amenities; ii) household structure, including distribution of age, sex, and employment of household members; iii) food expenditure and security; iv) costs of engaging with TB care including direct costs (e.g. medicines, clinic visits, food, and travel) and indirect costs (e.g. lost income), which will be evaluated using an adapted version of WHO’s TB Patient Costs Survey integrated into the interview ^22, 23^; v) coping strategies including dissaving (e.g. selling assets), schooldays lost, and temporary income-generating activities; vi) TB-related knowledge including understanding of transmission, prevention, and treatment of TB; vii) psychosocial situation evaluated through questions relating to social capital, quality of life, and stigma (Controls without TB will not be asked any questions about the impact that having TB disease has on their psychosocial situation); and viii) access and uptake of existing social protection schemes (whether TB-specific or TB-inclusive) and support for TB-affected households, evaluated through use of both closed ranking and open free-text questions to establish what socioeconomic and other support people with TB and their households receive or would like to receive.

Prior to implementation of household visits, the questionnaire will be translated from English into Nepali and then back-translated into English. It will be assessed by members of the study team and BNMT implementation staff before being piloted in approximately 10 patients. The questionnaire may subsequently be refined and questions deleted or added, depending on the pilot outcomes.

In addition to the above, Cases’ TB treatment outcomes will be collated from NTP and IMPACT-TB data and their association with interview responses analysed. This will provide an exploratory analysis of the association of socioeconomic position, socioeconomic impact of having TB, and TB treatment outcomes. A comparison of the socioeconomic position of households of Cases and Controls will also be made.

**Figure 3.**
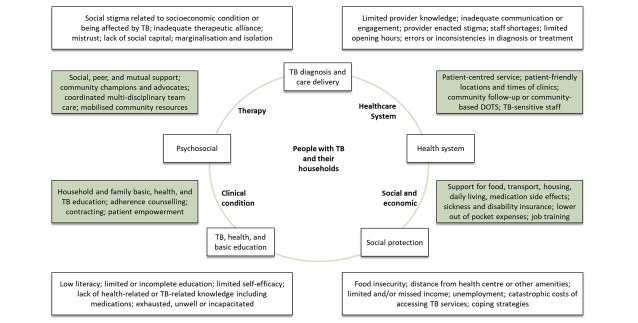
Conceptual framework: the barriers and facilitators to TB diagnosis and care The central circle, which contains dimensions of medication adherence adapted from the World Health Organization ^32^, is surrounded by the five main categories of relevant potential interventions. The factors that may promote access to and engagement with TB services (facilitators) are shown in green boxes and factors that may threaten access and engagement (barriers) are shown in white boxes. Therapeutic alliance refers to strong provider-patient relationships. Although Project 2 would gather data on all five dimensions, the psychosocial and social protection dimensions were perceived by the project team to be most pertinent to development of the socioeconomic intervention and were selected for further focus and discussion during Project 2’s workshop.

Project 2 will use and develop mixed methods research techniques ^10– 15^ to conduct a pre-FGD survey, FGDs, and workshop with approximately 40 key stakeholders in Nepal to identify the socioeconomic impact, barriers and facilitators to accessing and engaging with TB diagnosis and care. To inform the design of the FGDs and workshop, we created a conceptual framework for the barriers and facilitators to TB diagnosis and care, which was adapted from a World Health Organisation framework for medication adherence ( Figure 3) ^33^.

A short individual pre-FGD survey (see Project 2 Survey, *Extended data*) ^29^ will be provided to participants prior to the initiation of an FGD. The survey will detail: participants’ demographics; the stakeholder group to which the participant belongs; and their opinions on community, patient, health system, existing social protection schemes, and wider obstacles to achieving successful TB treatment outcomes.

The FGDs will be semi-structured and incorporate open-ended questions concerning barriers and facilitators to accessing and engaging with TB care in Nepal, and existing platforms and potential opportunities to mitigate these barriers including social protection schemes (see Project 2 Focus Groups, *Extended data*) ^29^. The FGDs will be conducted with separate groups of approximately eight key stakeholders. Stakeholders will be invited to participate according to their background (e.g. people with TB will be asked to participate in one FGD, and TB healthcare professionals will be asked to participate in another separate FGD). Towards the end of each FGD, participants will be asked to privately rank the top three most important barriers or facilitators to accessing TB diagnosis and care identified by the group during the FGD, and these responses will be collated for each FGD and across FGDs. The FGDs will be moderated by members of the project team trained in qualitative methods including conducting FGDs. The discussions will be audio recorded in Nepali language, translated into English, and back-translated by a translator who is not part of the project team. Each FGD group will be asked to elect a representative to feed their group’s outputs back at the subsequent workshop.

The final activity in Project 2 will be a one-day workshop bringing together the 40 key stakeholders (see Project 2 Workshop, *Extended data*) ^29^. The morning section of the workshop will consist of interactive presentations from the project team and stakeholder group representatives (including leaders of national social protection schemes in Nepal), and discussions exploring and validating the barriers and opportunities identified during the pre-FGD survey and interviews during FGDs. The afternoon section of the workshop will consist of multi-sectoral working groups (≤10 diverse stakeholders) developing a shortlist of potential socioeconomic interventions for TB-affected households in Nepal. The shortlist is not intended to consist of defined, unalterable packages, which are immediately ready for trial implementation. Rather, the potential interventions selected are intended to consist of what stakeholders perceive to be key elements or ingredients of psychosocial and economic support for TB-affected households, which are feasible and locally-appropriate to the Nepalese context. The interventions will be presented to the group including strengths, weaknesses, and potential sources of funding for implementation.

**Figure 4.**
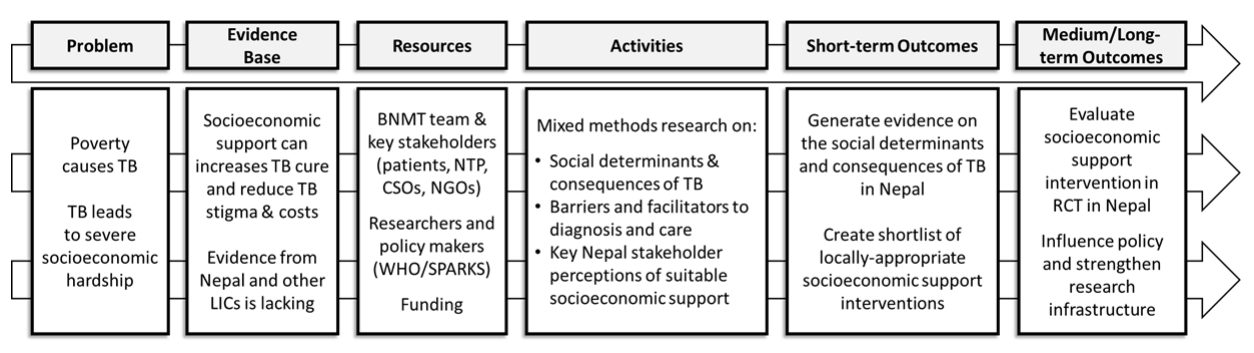
Logic model for research in Nepal to develop a locally-appropriate socioeconomic support intervention for TB-affected households. Abbreviations: CSOs = Civil Society Organisations; NGOs = Non-governmental organisations; NTP = National Tuberculosis Programme; WHO = World Health Organisation; SPARKS = Social Protection Action Research and Knowledge Sharing network ( www.sparks.ki.se)

The activities involved in this research form part of a process to develop and evaluate a complex, socioeconomic support intervention. In line with the Medical Research Council’s guidance on process evaluation of complex interventions, we developed a logic model to illustrate the developmental stages of the intervention ( Figure 4) ^33^.

This Wellcome research will aim to achieve the short-term outcomes described in the logic model. If follow-on funding is successfully obtained, the process to develop and implement the intervention and achieve the long-term outcomes will continue beyond this Wellcome research. This will include adaptation, piloting, and then large-scale randomised trial evaluation of the refined intervention.

### Outcomes to be measured

This exploratory Wellcome Trust funded Seed Award in Science research in four districts of Nepal will:

i. characterise social determinants of TB by comparing poverty level, education level, food security, and other socioeconomic factors of people with TB (Cases) versus people without TB (Controls);
ii. provide new insight into the barriers, facilitators, and socioeconomic impact of being ill with TB and accessing TB diagnosis and care, and compare this in Cases diagnosed by ACF versus PCF; and
iii. generate a community-led shortlist of the most feasible, equitable, and locally-appropriate socioeconomic interventions for TB-affected households to mitigate the socioeconomic impact of TB.

### Data collection and management

During the implementation of Project 1, information will be collected by BNMT district coordinators, community volunteers, and community mobilisers with support from the project team during visits to recruited patient households. This information includes but is not limited to socioeconomic, health, psychosocial, and behavioural data. This data will be collected on paper due to digital collection (e.g. on tablets or mobile phones) having issues with security and feasibility. The data of consenting TB patients will subsequently be linked with data from NTP’s TB patient register as part of its routine surveillance data collection at the intervention and control areas, with pre-existing permission from the NTP.

During Project 2, key stakeholders identified by the scoping exercise will complete a short pre-FGD survey in person, participate in an FGD, and participate in a one-day workshop.

All paper-based copies, including medical records, informed consent forms and participant information leaflets, will contain only a unique study identifier for each participant. These documents will be stored in a locked room in the BNMT office. Data will be checked for consistency and completeness by the project manager and double-checked by the PI prior to entering into an encrypted access database. The database will be managed by the data management team at KNCV TB Foundation in The Netherlands in line with data collected during the IMPACT-TB project. The data will be protected by KNCV on a password-secured server with availability limited to only key members of the study team when required for analysis.

### Data analysis and statistical plan

The quantitative data collected during the household visits of Project 1 will be analysed using simple descriptive statistics. Continuous costs data will be summarised by their arithmetic means and their 95% confidence intervals whether the data is Gaussian or non-Gaussian, because this approach is considered to be robust for health economics data analysis ^6, 34– 36^. Furthermore, because of the skewed nature of some expenditure data, median values may be zero or close to zero limiting the descriptive usefulness of presenting median values. As described in the PI’s previous research ^6^, any direct expenses, lost income, or annual income recorded as “zero” or missing will be replaced with the mean cost of each costs category, i.e. mean direct costs or lost income. The local currency, Nepalese rupee, will be converted into United States Dollars (conversion rate and date estimated through Oanda at time of data collection). Categorical data will be summarised as proportions with 95% confidence intervals.

With regards to analysis of household income and expenditure, the WHO TB Patients Costs Survey methods will be followed. In order to evaluate the optimal analytical strategy for costs data in Nepal, the analysis will compare and contrast the different approaches used in the WHO Survey to estimate household income (self-reported household consumption; self-reported household expenditure; self-reported household income; and estimated income based on household asset ownership and dwelling characteristics) and lost income (output approach of reported income pre- and during TB versus human capital approach of multiplying hours of work lost by hourly rate or daily in rate in cases of hospitalised patients).

Social determinants of TB including sociodemographic characteristics, socioeconomic position, stigma and social capital levels, and TB-related knowledge will be compared between ACF patients, PCF patients, and controls using Chi-squared test, Pearson’s test, one-way ANOVA, and multiple logistic regression models where appropriate. No comparison will be made between patients and controls concerning healthcare expenditure as this data was not collected from controls. Statistical analysis will be performed using the statistical software package STATA v13.1 (Statacorp, TX, USA).

The Framework method of thematic analysis will be used to manage and analyse data from Project 2 via the NVivo qualitative software package (Version 12) as per published social policy and tuberculosis research ^21– 24^. Specifically, two researchers (KD and TW) will familiarize themselves with the data through successive reading of transcripts; use both open and closed first order data coding to label data within NVivo; group codes together into a second order codebook of themes and sub-themes. Themes and subthemes will then be further stratified by third order coding to the level at which they predominantly occurred (e.g. individual or household level, health systems level, community level, or governmental level). The primary approach to codes, themes, and sub-themes will be deductive (top down), being informed by our conceptual framework and preliminary analysis of data from Project 1 relating to the social determinants and consequences identified. As coding continues, a secondary approach will integrate inductive (bottom up) coding in order to be iterative, responsive, and flexible as further data becomes available and is collated following each successive FGD.

### Plans for dissemination of study findings

The intended research outputs of this work are to: i) present the interim and final findings at the International Union Against TB and Lung Disease in October 2019 and October 2020, respectively; ii) publish, by June 2020, at least two papers in high-impact, peer-reviewed journals concerning the socioeconomic impact of accessing TB care in Nepal and the collaborative development of a shortlist of locally-appropriate socioeconomic interventions in Nepal; iii) feedback findings to the IMPACT-TB team, key stakeholders (including NTP and TB civil-society), the SPARKS (Social Protection Action Research and Knowledge Sharing) network, and WHO; iv) consolidate a close collaboration, good working relationship, and strong research infrastructure between BNMT and the NTP; and iv) to develop the protocol for a robust, large-scale randomised controlled trial to evaluate socioeconomic support for TB-affected households using the evidence generated by this mixed-methods study.

### Study status

Data collection for Project 1 during household visits began in May 2018 and is nearly complete at the time of writing with only TB treatment outcome data still being collected. Data collection for Project 2 during pre-FGD surveys and FGDs began in August 2018 and was completed in July 2019. The national workshop with key stakeholders was conducted on 11 and 12 September 2019 in Kathmandu, Nepal.

Data analysis, write-up, and dissemination of findings will begin in February 2020.

## Discussion

The overarching pledge of the Sustainable Development Goals (SDGs) is to “leave no one behind”. In 2018, over 3 million people with tuberculosis (TB) were not diagnosed, not notified, or their quality of care was unknown. In the same year, 1.5 million people with TB died and nearly a fifth of people diagnosed with TB did not have a successful treatment outcome ^16^. While millions of people with TB continue to be left behind, the SDG pledge is far from being realised.

Despite renewed interest in addressing social determinants of tuberculosis, there remain stark global inequalities in disease burden and access to TB care ^8^. At a population level, LICs bear the highest TB prevalence ^16^. At an individual level, people with TB are often vulnerable, impoverished, and their households suffer disproportionate financial shock due to their illness ^6, 13^. To eliminate such gross disparity, SDG slogans must be turned into actions.

WHO’s 2015 End TB Strategy acknowledges the need to reduce inequalities in TB prevention and care. A key component of Pillar 2 (Bold policies and supportive systems) of the strategy is social protection and poverty alleviation to reduce catastrophic costs of TB-affected households and improve TB outcomes ^2^. However, there is minimal evidence to guide this policy change.

This mixed-methods will generate evidence concerning the socioeconomic position of TB-affected households, the impact that having TB disease has on that position and explore the coping strategies that households use to mitigate the impact of the disease. Moreover, this research will: use methods to measure costs of TB-affected households to which members of the study team contributed as part of the WHO Task Force on Catastrophic Costs of Tuberculosis; and provide the first known comparison of the socioeconomic impact of TB on people with TB diagnosed through PCF versus ACF. The case-control element of Project 1 will allow comparison of the sociodemographic characteristics, socioeconomic position, stigma and social capital levels, and TB-related knowledge between people with TB (Cases) and people without TB (Controls).

The further significance of the study lies in its development of a shortlist of a locally-generated intervention to provide socioeconomic support to TB-affected households. Through collaboration with diverse stakeholders in Nepal from patients to NTP managers to civil-society representatives, it is hoped that the shortlist created will feature interventions that are both locally-appropriate and feasible. Furthermore, this tight collaboration should aid design and implementation of a larger, randomised-controlled trial, and also future translation of research findings into national policy in Nepal. Beyond the national impact, these findings and those of the future trial will also offer evidence for scale-up of socioeconomic support in other resource-limited countries with a high TB burden. Complementary to pills and tests, this socioeconomic support will be an essential part of eliminating TB by 2050.

## Conclusions

This mixed-methods study will fill this existing evidence gap by examining the costs of accessing TB care at a household level through longitudinal application of an adapted WHO TB Patient Costs Survey throughout TB treatment. This data will be enriched by collecting complementary data on household socioeconomic position, coping strategies, food insecurity, TB knowledge, social capital, and quality of life. The findings will then feed into a short survey and semi-structured FGDs with key stakeholders in Nepal to consider, at a local, regional, and national level, what are the leading barriers and facilitators to accessing and engaging in TB care, and what might be the most locally-appropriate interventions to address the socioeconomic impact of having TB disease.

## Data availability

### Underlying data

No data are associated with this article

### Extended data

Open Science Framework: Research protocol for a mixed-methods study to characterise and address the socioeconomic impact of accessing TB diagnosis and care in Nepal. https://doi.org/10.17605/OSF.IO/6TC4F ^29^

This project contains the following extended data:

- Final_Wellcome Seed Award Project 1 Patient Interview v8.pdf
- Final Patient _Wellcome Seed Award Project 1 Consent Form NEPALI.pdf
- Final Patient _Wellcome Seed Award Project 1 Interview v8 NEPALI.pdf
- Final Patient _Wellcome Seed Award Project 1 Patient Info Leaflet NEPALI.pdf
- Wellcome Seed Award Project 1 Patient Consent Form.pdf
- Wellcome Seed Award Project 1 Patient Information Leaflet ENGLISH.pdf
- Wellcome Seed Award Project 2 Survey and Focus Group Consent Form.pdf
- Wellcome Seed Award Project 2 Survey and Focus Group Participant Information Leaflet.pdf
- Wellcome Seed Award Project 2 Survey v4 20180525.pdf
- Wellcome Seed Award Project 2 Workshop Consent Form.pdf
- Wellcome Seed Award Project 2 Workshop Participant Information Leaflet.pdf

Data are available under the terms of the Creative Commons Zero "No rights reserved" data waiver (CC0 1.0 Public domain dedication).
