# Supplementary material for: Research protocol for a mixed-methods study to characterise and address the socioeconomic impact of accessing TB diagnosis and care in Nepal
Source: Wellcome Open Res. 2020 Jun 16;5:19. Originally published 2020 Feb 5. [Version 2] doi: 10.12688/wellcomeopenres.15677.2 (PMC7489278; doi:10.12688/wellcomeopenres.15677.2)
Supplement: Peer review history

# Peer review: Reviewer response for version 2

**DOI:** 10.21956/wellcomeopenres.17629.r39129

**Published:** 2020-09-11

**Authors:** Delia Boccia

**Affiliations:** 1 Faculty of Public Health and Policy, London School of Hygiene and Tropical Medicine, London, UK

I confirm that the authors have appropriately and convincingly addressed all my comments.

Is the study design appropriate for the research question?

Partly

Is the rationale for, and objectives of, the study clearly described?

Yes

Are sufficient details of the methods provided to allow replication by others?

Partly

Are the datasets clearly presented in a useable and accessible format?

Not applicable

Reviewer Expertise:

Social determinants of TB, social protection.

I confirm that I have read this submission and believe that I have an appropriate level of expertise to confirm that it is of an acceptable scientific standard.

# Peer review: Reviewer response for version 2

**DOI:** 10.21956/wellcomeopenres.17629.r39128

**Published:** 2020-08-07

**Authors:** Priya B. Shete

**Affiliations:** 1 University of California San Francisco, San Francisco, CA, USA

No further comments. I appreciate the authors attention to my previous suggestions and feel that they have taken care to improve the paper with these details.

Is the study design appropriate for the research question?

Yes

Is the rationale for, and objectives of, the study clearly described?

Yes

Are sufficient details of the methods provided to allow replication by others?

Partly

Are the datasets clearly presented in a useable and accessible format?

Not applicable

Reviewer Expertise:

Implementations science, tuberculosis, social protection.

I confirm that I have read this submission and believe that I have an appropriate level of expertise to confirm that it is of an acceptable scientific standard.

# Peer review: Reviewer response for version 1

**DOI:** 10.21956/wellcomeopenres.17181.r37807

**Published:** 2020-02-26

**Authors:** Priya B. Shete

**Affiliations:** 1 University of California San Francisco, San Francisco, CA, USA

The major area for improvement with this protocol would be more discussion in the methods about how the qualitative data will be coded and subsequently used to then generate a menu of feasible options for pilot implementation and evaluation to overcome targeted barriers. I would appreciate further discussion of the conceptual framework that informs the data collection tools/interview guides in Project 2. As Project 2 is particularly important for identifying targeted barriers and facilitators of care for future interventions, I would also like to see what deterministic framework or model is being used to develop those interventions. Any reference to frameworks from implementation science or social epidemiology would perhaps be helpful.

Is the study design appropriate for the research question?

Yes

Is the rationale for, and objectives of, the study clearly described?

Yes

Are sufficient details of the methods provided to allow replication by others?

Partly

Are the datasets clearly presented in a useable and accessible format?

Not applicable

Reviewer Expertise:

Implementations science, tuberculosis, social protection.

I confirm that I have read this submission and believe that I have an appropriate level of expertise to confirm that it is of an acceptable scientific standard, however I have significant reservations, as outlined above.

# Response

**Published:** 2020-06-11

**Authors:** Tom Wingfield

**Affiliations:** Liverpool School of Tropical Medicine, UK

***We are indebted to Reviewer 2 for these excellent comments, which we have addressed and which we now believe serve to strengthen the protocol. It is important to note that this Wellcome Trust funded study began in 2018 and data collection had already been completed prior to submission of the protocol to Wellcome Open Research. When the research team became aware of Wellcome Open Research, we approached the editorial team who advised that it was still appropriate to submit a study protocol despite data collection being complete. While this is important for critical peer review of the protocol it does, unfortunately, mean that we are unable to adjust parts of the protocol that have been completed and written-up including, for example, the coding strategy.***

***However, we were able to address Reviewer 2’s comments by providing further detail about the coding techniques used and necessary clarification about how the quantitative and qualitative data fed informed outline of the design of the socioeconomic support interventions. In addition, we omitted to include the conceptual framework and logic model that we employed for this research, which we have now rectified and, again, this has helped to improve the protocol and will also be included in subsequent, related publications. Thank you to the Reviewer for their constructive review.***

***Study activities section:*** ***“*** ***Project 2 will use and develop mixed methods research techniques ^10^^– ^^15^ to conduct a pre-FGD survey, FGDs, and workshop with approximately 40 key stakeholders in Nepal to identify the socioeconomic impact, barriers and facilitators to accessing and engaging with TB diagnosis and care. To inform the design of the FGDs and workshop, we created a conceptual framework for the barriers and facilitators to TB diagnosis and care, which was adapted from a World Health Organisation framework for medication adherence ( Figure 3). ^30^***

*(PLEASE SEE FIGURE IN UPDATED PROTOCOL)*

***Figure 3.*** ***Conceptual framework: the barriers and facilitators to TB diagnosis and care.***

***The central circle, which contains dimensions of medication adherence adapted from the World Health Organization*** ***^30^*** ***, is surrounded by the five main categories of relevant potential interventions. The factors that may promote access to and engagement with TB services (facilitators) are shown in green boxes and factors that may threaten access and engagement (barriers) are shown in white boxes. Therapeutic alliance refers to strong provider-patient relationships. Although Project 2 would gather data on all five dimensions, the psychosocial and social protection dimensions were perceived by the project team to be most pertinent to development of the socioeconomic intervention and were selected for further focus and discussion during Project 2’s workshop.”***

***Study activities section:*** ***“*** ***The final activity in Project 2 will be a one-day workshop bringing together the 40 key stakeholders (see Project 2 Workshop, Extended data)*** ***^29^*** ***. The morning section of the workshop will consist of interactive presentations from the project team and stakeholder group representatives (including leaders of national social protection schemes in Nepal), and discussions exploring and validating the barriers and opportunities identified during the pre-FGD survey and interviews during FGDs.*** ***The afternoon section of the workshop will consist of multi-sectoral working groups (≤10 diverse stakeholders) developing a shortlist of potential socioeconomic interventions for TB-affected households in Nepal. The shortlist is not intended to consist of defined, unalterable packages, which are immediately ready for trial implementation. Rather, the potential interventions selected are intended to consist of what stakeholders perceive to be key elements or ingredients of psychosocial and economic support for TB-affected households, which are feasible and locally-appropriate to the Nepalese context. The interventions will be presented to the group including strengths, weaknesses, and potential sources of funding for implementation.***

***The activities involved in this research form part of a process to develop and evaluate a complex, socioeconomic support intervention. In line with the Medical Research Council’s guidance on process evaluation of complex interventions, we developed a logic model to illustrate the developmental stages of the intervention ( Figure 4). ^31^***

*(PLEASE SEE FIGURE IN UPDATED PROTOCOL)*

***Figure 4. Logic model for research in Nepal to develop a locally-appropriate socioeconomic support intervention for TB-affected households.***

***This Wellcome research will aim to achieve the short-term outcomes described in the logic model. If follow-on funding is successfully obtained, the process to develop and implement the intervention and achieve the long-term outcomes will continue beyond this Wellcome research. This will include adaptation, piloting, and then large-scale randomised trial evaluation of the refined intervention.”***

***Data analysis and statistical plan section:*** ***“*** ***The Framework method of thematic analysis will be used to manage and analyse data from Project 2 via the NVivo qualitative software package (Version 12) as per published social policy and tuberculosis research ^21^^– ^^24^ . Specifically, two researchers (KD and TW) will familiarize themselves with the data through successive reading of transcripts; use both open and closed first order data coding to label data within NVivo; group codes together into a second order codebook of themes and sub-themes. Themes and subthemes will then be further stratified by third order coding to the level at which they predominantly occurred (e.g. individual or household level, health systems level, community level, or governmental level). The primary approach to codes, themes, and sub-themes will be deductive (top down), being informed by our conceptual framework and preliminary analysis of data from Project 1 relating to the social determinants and consequences identified. As coding continues, a secondary approach will integrate inductive (bottom up) coding in order to be iterative, responsive, and flexible as further data becomes available and is collated following each successive FGD.”***

# Peer review: Reviewer response for version 1

**DOI:** 10.21956/wellcomeopenres.17181.r37826

**Published:** 2020-02-21

**Authors:** Delia Boccia

**Affiliations:** 1 Faculty of Public Health and Policy, London School of Hygiene and Tropical Medicine, London, UK

This paper describes in detail the protocol of a study aiming at understanding better the social determinants of TB as well as the financial consequences of TB. The study is very relevant: it is epidemiologically sound and the rational for it is clear and strongly supported by the existing literature.

 There are two gaps that – in my opinion – should be better filled to make the protocol stronger:

1. The selection of controls for the case-control study. This requires a further dissertation about how practically controls will be selected and how the authors are planning to minimise selection biases. Authors explain why an age-sex matching approach could not be adopted; however, the description of the selection of controls remain elusive which triggers questions like: what sampling strategy will be adopted? How randomness as well as representativeness will be respected? General population controls may be a good option for answering questions around the social determinants of TB; however, I am not convinced they are also the best choice when looking at catastrophic health expenditures. Obviously healthy controls will be better off and wonder whether this second research question could be not better addressed by selecting hospital controls or among people who are ill-affected (albeit not from TB). I suggest the authors to better justify their choices and discuss why alternative options have been dismissed.
2. The qualitative work should include a stronger effort to address not just barriers to access to TB care, but also barriers to existing social protection schemes experienced by TB-patients. It feels like a gigantic missed opportunity if not embedded into the existing plans. If indeed the authors are planning to ascertain existing social protection options for TB patients and access can be maximised, then this should be explicitly stated in the paper.

Is the study design appropriate for the research question?

Partly

Is the rationale for, and objectives of, the study clearly described?

Yes

Are sufficient details of the methods provided to allow replication by others?

Partly

Are the datasets clearly presented in a useable and accessible format?

Not applicable

Reviewer Expertise:

Social determinants of TB, social protection.

I confirm that I have read this submission and believe that I have an appropriate level of expertise to confirm that it is of an acceptable scientific standard, however I have significant reservations, as outlined above.

# Response

**Published:** 2020-06-11

**Authors:** Tom Wingfield

**Affiliations:** Liverpool School of Tropical Medicine, UK

1. The selection of controls for the case-control study. This requires a further dissertation about how practically controls will be selected and how the authors are planning to minimise selection biases. Authors explain why an age-sex matching approach could not be adopted; however, the description of the selection of controls remain elusive which triggers questions like: what sampling strategy will be adopted? How randomness as well as representativeness will be respected? General population controls may be a good option for answering questions around the social determinants of TB; however, I am not convinced they are also the best choice when looking at catastrophic health expenditures. Obviously healthy controls will be better off and wonder whether this second research question could be not better addressed by selecting hospital controls or among people who are ill-affected (albeit not from TB). I suggest the authors to better justify their choices and discuss why alternative options have been dismissed.

***Author response:*** ***Thank you for raising this important issue with which we wholeheartedly agree. It is important to note that this Wellcome Trust funded study began in 2018 and data collection had already been completed prior to submission of the protocol to Wellcome Open Research. When the research team became aware of Wellcome Open Research, we approached the editorial team who advised that it was still appropriate to submit a study protocol despite data collection being complete. While this is important for critical peer review of the protocol it does, unfortunately, mean that we are unable to adjust the protocol including, for example, sampling strategies at this stage. However, we have attempted to respond to the reviewer’s comments about sampling strategy and control population randomness and representativeness. We have also made it clearer that the data from controls will only be used to characterise social determinants of TB and there will be no analysis comparing health expenditure in controls vs people with TB, which we agree would be inappropriate with general population controls.***

***Study population section:*** ***“*** ***Concurrently, 100 people without TB from the study sites (controls) will be invited to participate with written, informed consent (see Project 1 Interview Healthy Control Consent Form, Extended data)*** ***^29^*** ***. Due to constraints in study budget, duration, and field logistics, it will not be possible to age and sex match cases and controls nor to randomly select controls using geospatial or other household randomisation techniques. Therefore, a convenience sampling strategy was used.*** ***In order to be as widely representative of and generalisable to the background population as possible, following project team and community mobiliser discussions in the study sites, we opted to recruit control participants from diverse locations within the districts, which were attended by a broad demographic cross-section of the local population in terms of age, gender, and socioeconomic position.*** ***These diverse study site locations include: tea houses, primary healthcare centres, antenatal and immunization clinics, door-to-door visits following sputum camps (e.g. people who tested negative for TB), and public gathering places.  Interviewers will visit the control recruitment locations at similar times of the morning and aim to consecutively recruit all individuals in attendance at that location. Similar numbers of participants from each recruitment location will be recruited until the sample size of 100 people without TB is reached. We acknowledge that convenience sampling may be associated with a higher likelihood of a non-generalisable control cohort than other techniques. To try to address this, the survey used to collect data from the controls has incorporated multiple questions concerning sociodemographic variables, including education level, occupation, amenities, and assets, from the most recent version of the Nepal Household Survey, which is publicly available. Descriptive analysis will evaluate whether the controls recruited are representative of the wider population in those districts through comparison of their sociodemographic data with respondents to the national survey in the same districts. Any differences between the control population and background population will then be highlighted transparently in corresponding research outputs and publications.”***

***“Data analysis and statistical plan section:*** ***“*** ***Social determinants of TB including sociodemographic characteristics, socioeconomic position, stigma and social capital levels, and TB-related knowledge will be compared between ACF patients, PCF patients, and controls using Chi-squared test, Pearson’s test, one-way ANOVA, and multiple logistic regression models where appropriate. No comparison will be made between patients and controls concerning healthcare expenditure as this data was not collected from controls.”***

2. The qualitative work should include a stronger effort to address not just barriers to access to TB care, but also barriers to existing social protection schemes experienced by TB-patients. It feels like a gigantic missed opportunity if not embedded into the existing plans. If indeed the authors are planning to ascertain existing social protection options for TB patients and access can be maximised, then this should be explicitly stated in the paper.

***Author Response: We agree entirely with the reviewer and are grateful for this apposite point. Within the confines of not being able to alter the protocol given that data collection is now completed, we have clarified this important issue in the protocol to explain that, across Projects 1 and 2, quantitative and qualitative data concerning social protection in Nepal was gathered, including the coverage, target recipients, barriers to access, and limitations of existing packages.***

***Study activities section:*** “ ***The interviews will be structured (see Project 1 Interview, Extended data)*** ***^29^*** ***and gather data on: i) socioeconomic position, evaluated by a multi-dimensional poverty score*** ***^6^*** ***^,^*** ***^9^*** ***^,^*** ***^13^*** ***^,^*** ***^15^*** ***assessing dwelling characteristics, assets, and access to amenities; ii) household structure, including distribution of age, sex, and employment of household members; iii) food expenditure and security; iv) costs of engaging with TB care including direct costs (e.g. medicines, clinic visits, food, and travel) and indirect costs (e.g. lost income), which will be evaluated using an adapted version of WHO’s TB Patient Costs Survey integrated into the interview*** ***^22^*** ***^,^*** ***^23^*** ***; v) coping strategies including dissaving (e.g. selling assets), schooldays lost, and temporary income-generating activities; vi) TB-related knowledge including understanding of transmission, prevention, and treatment of TB; vii) psychosocial situation evaluated through questions relating to social capital, quality of life, and stigma (Controls without TB will not be asked any questions about the impact that having TB disease has on their psychosocial situation); and viii) access and uptake of existing social protection schemes (whether TB-specific or TB-inclusive) and support for TB-affected households, evaluated through use of both closed ranking and open free-text questions to establish what socioeconomic and other support people with TB and their households receive or would like to receive.”***

***Study activities section:*** ***“*** ***A short individual pre-FGD survey (see Project 2 Survey, Extended data)*** ***^29^*** ***will be provided to participants prior to the initiation of an FGD. The survey will detail: participants’ demographics; the stakeholder group to which the participant belongs; and their opinions on community, patient, health system, existing social protection schemes, and wider obstacles to achieving successful TB treatment outcomes.***

***The FGDs will be semi-structured and incorporate open-ended questions concerning barriers and facilitators to accessing and engaging with TB care in Nepal, and existing platforms and potential opportunities to mitigate these barriers including social protection schemes (see Project 2 Focus Groups, Extended data)*** ***^29^*** ***.***

***Study activities section:*** ***“*** ***The final activity in Project 2 will be a one-day workshop bringing together the 40 key stakeholders (see Project 2 Workshop, Extended data)*** ***^29^*** ***. The morning section of the workshop will consist of interactive presentations from the project team and stakeholder group representatives (including leaders of national social protection schemes in Nepal), and discussions exploring and validating the barriers and opportunities identified during the pre-FGD survey and interviews during FGDs.*** ***”***
